# Campylobacteriosis outbreak associated with consumption of undercooked chicken liver pâté in the East of England, September 2011: identification of a dose–response risk

**DOI:** 10.1017/S0950268813001222

**Published:** 2013-05-28

**Authors:** D. S. EDWARDS, L. M. MILNE, K. MORROW, P. SHERIDAN, N. Q. VERLANDER, R. MULLA, J. F. RICHARDSON, A. PENDER, M. LILLEY, M. REACHER

**Affiliations:** 1 Health Protection Agency (HPA) East of England Regional Epidemiology Unit (REU), Institute of Public Health, University Forvie Site, Cambridge, UK; 2 HPA Bedfordshire and Hertfordshire Health Protection Unit, Beacon House, Letchworth, Hertfordshire, UK; 3 HPA Statistics Unit, Statistics, Modelling and Economics Department of Health Protection Services, Colindale, London, UK; 4 Department of Medical Microbiology, Luton and Dunstable University Hospital; 5 GBRU, HPA Colindale, London, UK; 6 Housing and Public Protection Service, North Hertfordshire District Council, Herts, UK

**Keywords:** *Campylobacter*, foodborne zoonoses, outbreaks, public health

## Abstract

A foodborne outbreak with 49 cases (22 culture positive for *Campylobacter* sp.) following a wedding party in the East of England was investigated. A retrospective cohort study identified an association between consumption of chicken liver pâté and infection with *Campylobacter jejuni/coli*. There was a statistically significant association between dose (amount of chicken liver pâté eaten) and the risk of disease [‘tasted’: odds ratio (OR) 1·5, 95% confidence interval (CI) 0·04–∞; ‘partly eaten’: OR 8·4, 95% CI 1·4–87·5; ‘most or all eaten’: OR 36·1, 95% CI 3·3–2119). The local authority found evidence that the preparation of chicken livers breached Food Standards Agency's guidelines. This epidemiological investigation established a clear dose–response relationship between consumption of chicken liver pâté and the risk of infection with *Campylobacter*. The continuing need to raise public awareness of the risk to human health posed by undercooked chicken liver is evident.

## INTRODUCTION

UK outbreaks of campylobacteriosis associated with chicken liver dishes, in particular chicken liver pâté, increased substantially between 2009 and 2011 [1–5]. Campylobacteriosis is an acute bacterial enteric disease following infection with *Campylobacter* sp. usually *Campylobacter jejuni*. Onset following exposure is commonly 2–5 days, but can be up to 10 days. Symptoms of campylobacteriosis include malaise, fever, and severe abdominal pain often many hours before onset of diarrhoea (sometimes bloody), nausea, and/or vomiting [6]. The duration of this debilitating gastrointestinal disease is commonly 1–7 days, but can be longer, and is, in the majority of cases, self-limiting [6].

An outbreak of gastroenteritis (diarrhoea and/or vomiting) was reported to the Health Protection Agency (HPA) Bedfordshire and Hertfordshire Health Protection Unit (HPU) on 8 September 2011 by a local authority environmental health officer (EHO). This followed notification to the local authority on 8 September 2011 by a member of the public that several people were reporting illness, including diarrhoea and vomiting, after attending a wedding on 3 September. The wedding party included a catered event with afternoon and evening meals.

The HPU formed an outbreak control team which decided to conduct an analytical investigation and, on the same day, notified the HPA East of England Regional Epidemiology Unit (REU) for support with study design and analysis. This paper provides a summary of an analytical study conducted to test the hypothesis that consumption of chicken liver pâté at the wedding party was significantly associated with developing gastrointestinal illness. The results of the local authority investigation are also included, as this both informed the design of the epidemiological investigation and the interpretation of the findings.

## METHODS

### Study design and cohort

A retrospective cohort study was used which involved guests attending a wedding party on 3 September 2011 (the exposed). The cohort was defined as guests who ate at the wedding afternoon meal and/or evening meal (*N* = 118).

### Questionnaire content

The REU developed a questionnaire suitable for an event with two separate catered meals. The questions included personal details, date of onset and symptoms of illness, details on contact with health professionals, illness in household members who did not attend the wedding party, travel history, food and drink consumed at the wedding afternoon meal and/or the evening meal. The menu for the wedding party afternoon meal and evening meal was obtained from the caterer and used to inform the questionnaire.

### Data collection

Questionnaires were sent by post via the HPU to all guests who attended the wedding party. Returned questionnaires were double-data entered and validated using EpiData computer software (Epidata Association, Denmark) [7]. Following preliminary data cleaning and analysis in EpiData analysis an anonymized dataset was sent to the HPA Statistics Unit Health Protection Services for definitive statistical analysis.

### Case definition

A case was defined as an individual who ate or drank during one or both of the wedding party meals (wedding afternoon meal or evening meal) on 3 September 2011 and who reported illness with diarrhoea (defined as ⩾3 loose stools in a 24-h period) or vomiting with or without other gastrointestinal symptoms by 8 September 2011. Respondents (cases and non-cases) were excluded if they had travelled abroad in the 5 days prior to attending the wedding or if other people in their household, who did not attend the wedding, had suffered from a gastrointestinal illness within a week after the wedding, whether or not the wedding guest was the first to become ill.

### Data analysis

Univariable analysis was performed using EpiData Analysis 2.2.1 (Epidata Association) and Stata v. 11.2 (StataCorp., USA), with tabular and regression methods including Fisher's exact test for dichotomous variables and likelihood ratio testing for variables with more than two categories [7, 8]. The multivariable analysis was performed using Stata v. 11.2 with initial multivariable logistic regression modelling, which included all exposure variables from the single variable analysis with elevated risk ratios and a *P* value of ⩽0·2, and age group and sex as potential confounders. A backwards stepwise procedure was used with the least significant variable removed at each step provided it was not a substantial confounder, but always retaining age and sex. The *P* values were determined by means of a likelihood ratio test. Any protective exposures that emerged were dropped from the model. Median unbiased estimates were calculated in exact logistic and Poisson regression models for those exposures with inestimable risk or odds ratios in the (asymptotic) regression models due to sampling zeros.

For some food items the amount that was consumed was quantified. To assess a dose–response relationship, the binary variable (consumption: yes/no) was replaced by a ‘dose’ variable with the categories, ‘none’, ‘tasted’, ‘partly eaten’ and ‘most or all eaten’. A sub-analysis of all non-cases and the *Campylobacter*-positive individuals (all of whom met the case definition) was conducted following the analysis procedure outlined above.

### Microbiological Investigation

Stool samples from 26 symptomatic guests were examined for a range of bacterial pathogens including *Campylobacter* sp. Putative *Campylobacter* isolates were identified by microaerophilic growth, oxidase-positivity and Gram stain in 22 samples. Thirteen patient isolates were submitted to the HPA reference laboratory for confirmation of identification and further characterization.

Isolates were received as swab culture, plated onto Columbia blood agar and incubated at 37°C for 48 h under microaerophilic conditions. *Campylobacter* sp. was grown from 12 samples. Purified cultures were speciated by real-time PCR, serotyped, phage-typed, and examined for antibiotic resistance [9–12].

## RESULTS

### Descriptive epidemiology

**Fig. 1.**
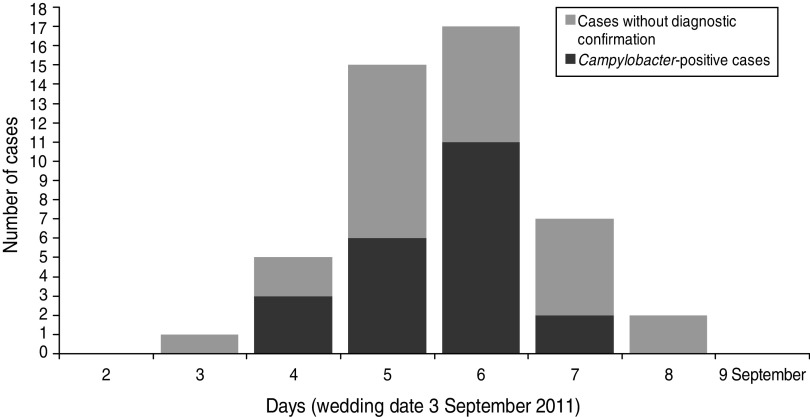
Epidemic curve of cases of gastrointestinal illness (including those identified as *Campylobacter* positive) following attendance at a wedding party in the East of England on 3 September 2011.

In total 102 (86·4%) questionnaires were returned with 49 respondents meeting the case definition. Three individuals were excluded from the analysis because they did not eat or drink at the wedding party. Two other individuals were excluded because household members who had not been wedding guests reported gastrointestinal symptoms. In total 97 responses were included in the analysis. There was no significant difference in demographic profiles between the cases and non-cases (Table 1). For 48 cases the earliest date of onset was late evening on the day of the wedding (at 23:00 hours) and the latest was 5 days later, with a peak of 17 cases 4 days later (Fig. 1). The shape of the epidemic curve indicates a point source consistent with a single exposure to *Campylobacter* through a food vehicle on a single day (Fig. 1). The mean incubation period for cases was 2·3 days (range 0–5 days, excluding one outlier).

**Table 1.** Demographic characteristics and symptoms of cases and non-cases of gastrointestinal illness following eating or drinking at wedding party on 3 September 2011

	Cases, *n* (%)	Non-cases, *n* (%)
Total respondents	49 (51)	48 (49)
Gender		
Females	26 (53)	23 (47)
Males	23 (49)	24 (51)
Gender unknown		1
Age		
Mean age (years)	39 years	40 years
⩽30	10 (43)	13 (57)
31–44	20 (48)	22 (52)
⩾45	19 (59)	13 (41)
Symptoms and medical attention (percentage of cases)		
Diarrhoea	45 (92)	
Loose stool	20 (41)	
Blood in stool	8 (16)	
Abdominal pain	48 (98)	
Fever	31 (63)	
Nausea	40 (82)	
Headache	35 (71)	
Vomiting	9 (18)	
Other symptom	19 (39)	
Visited GP	31 (63)	
Attended hospital	2 (4)	

The most frequently reported symptom was abdominal pain (98%), diarrhoea (92%) and vomiting (18%). Two thirds of cases (*n* = 31) reported that they had consulted a General Practitioner (GP) and 4% (*n* = 2) that they had attended hospital, although records indicate they were not admitted. Fifteen cases reported a date when symptoms ended, indicating a median duration of illness of 6 days (interquartile range 3–7 days, range 1–24 days).

### Microbiological results

Twenty-six individuals had a stool sample submitted for laboratory investigation, 22 of which were culture positive for *Campylobacter* sp. Four isolates of *Campylobacter* were identified as *C. coli* and eight as *C. jejuni*. The onset dates for cases that were culture test-positive for *Campylobacter* were days 2–5 after the wedding (Fig. 1).

### Univariable analysis

The univariable analysis identified statistically significant positive or negative associations (*P* < 0·05) and menu item variables which met inclusion criteria for multivariable analysis (positive association and *P* < 0·2). Association with illness was strongest [risk ratio (RR) 3·7, 95% confidence interval (CI) 1·6–8·8] for consumption of chicken liver pâté. Age group and sex were not significantly associated with developing gastrointestinal illness.

### Multivariable analysis

The multivariable logistic regression model included data from 66 individuals that had given responses to each of the exposures (Table 2). Chicken liver pâté was a significant risk factor (*P* < 0·001) with an odds ratio (OR) of 31·3 and 95% confidence interval (CI) of 6·1–161·1. The *P* value (<0·05) suggested that drinking vodka-based drinks may be a risk factor (OR 4·8, 95% CI 0·3–∞) but the estimated CI included 1 and was very wide indicating high uncertainty due to the small number of cases so the role of chance cannot be ruled out.

**Table 2.** Multivariable analysis with multi-categorical chicken liver pâté exposure variable for guests attending the afternoon meal at a wedding party in the East of England on 3 September 2011

	OR	95% CI	*P* value*
Age group (years)			
⩽30	1·0	Reference	0·8
31−44	1·0	0·1–22·2	
⩾45	0·5	0·02–11·4	
Sex			
Male	0·9	0·1–8·1	0·9
Female	1·0	Reference	
Chicken liver pâté			
None	1·0	Reference	<0·001
Tasted	1·5†	0·1–∞	
Partly eaten	8·4	1·4–87·5	
Most/all eaten	36·1	3·3–2119	
Vodka-based products 1 & 2			
Yes	4·4†	0·3–∞	0·03
No	1·0	Reference	
Soft drink or mixer			
Yes	4·7	0·5–45·5	0·2
No	1·0	Reference	
Coffee			
Yes	7·3	0·9–57·8	0·04
No	1·0	Reference	

OR, Odds ratio; CI, confidence interval.

† Median unbiased estimate.

* Likelihood ratio test.

A clear dose–response between the quantity of chicken liver pâté consumed and illness was found, although the 95% CIs overlapped between the categories ‘tasted’, ‘partly eaten’ or ‘eaten most or all’ (Table 2). This strengthens the case for a causal relationship between consumption of chicken liver pâté and gastrointestinal illness.

Univariable and multivariable logistic regression analysis restricted to cases that were culture-positive for *Campylobacter* sp. yielded similar results as analyses of all cases.

### Environmental assessment of catering by EHO

None of the pâté from the wedding or any other pâté was available for sampling. The kitchen at the wedding venue was found to be in good condition with evidence of effective cleaning and disinfection. The raw chicken livers used for the pâté were from a European Union (EU) country. The pâté was prepared by shallow frying chicken livers to retain the pink colour in their centre, the livers were then blended with a melted butter jus and the pâté was then pressed into a clingfilm-lined mould and blast-chilled to set. The record sheet indicated a core cooking temperature of 60°C for the chicken livers.

## DISCUSSION

The investigation showed that chicken liver pâté caused this outbreak of campylobacteriosis. The cohort study found a highly significant association with this food and no other and the environmental investigation showed that the chicken livers had been undercooked. The distribution of onset times and dates suggests that all cases occurred following exposure at the wedding party, with a mean incubation period of 2·3 days. This is consistent with the pattern of disease that would be expected for campylobacteriosis [5]. Due to the rapid response of the EHO and HPU a high proportion of the cases submitted stool samples from which the majority had a *Campylobacter* sp. isolated.

*Campylobacter* infection is the most common cause of gastrointestinal illness in the UK, with *C. jejuni* more frequently isolated than *C. coli*. The organism is often carried as a commensal organism in the gastrointestinal tract of poultry [13–15]. As a consequence *C. jejuni* or *C. coli* contaminate chicken meat and offal during the evisceration process post-slaughter and are able to survive subsequent refrigeration due to the relative moist environment that raw poultry meat and offal provides [16]. *C. jejuni* are present in the biliary tree of healthy birds leading to their presence within chicken livers even before slaughter occurs, making chicken liver an inherently hazardous food [17, 18]. A survey of *Campylobacter* spp. in UK retail poultry livers found *Campylobacter* strains isolated from the majority (69%) of chicken liver samples that were similar to strains commonly involved in human infection [19]. Similarly a survey of retail chicken livers and gizzards in Oklahoma, USA found 77% of 159 livers were contaminated with *Campylobacter* sp. (34% *C. jejuni* and 33% *C. coli*) [20].

Research in New Zealand on the effects of pan-frying chicken livers showed that inactivation of *Campylobacter* in chicken livers is proportional to cooking time [21]. The core temperatures of the livers stabilized at 70–80°C after 2·5 min of pan-fry cooking, but naturally occurring *C. jejuni* were not inactivated until 5-min cooking time. This highlights the importance of thorough cooking of poultry offal prior to consumption as a critical control point in the food chain to prevent human cases of campylobacteriosis. A critical factor in this reported wedding outbreak was the failure by the caterer to cook the chicken livers to a sufficient core temperature. The preparation method breached both the venue's food safety policy, which specified a core temperature of 75°C, as recommended by the Food Standards Agency (FSA), and the FSA guideline of a minimum core temperature of 65°C for 10 min.

The FSA highlighted the high risk posed by chicken livers with regard to a foodborne outbreak of campylobacteriosis in the first half of 2010, with chicken liver parfait and chicken liver pâté products associated with five outbreaks of campylobacteriosis [15]. The FSA issued guidance to caterers to ensure chicken liver is cooked through, with recommended core cooking times dependent on core temperatures [15]. In 2011 the HPA announced that 90% of outbreaks of foodborne outbreaks of campylobacteriosis at catering venues in 2011 were linked to chicken liver pâté consumption [22]. Chicken livers have been identified as a high-risk food product by the FSA, Environmental Health and Health Protection organizations in England. There is potential for improvement in the practices of caterers and the industry. Lapses have consequences for the public, in terms of preventable cases of foodborne campylobacteriosis, and catering businesses, which face serious impacts to their reputation and potential prosecution.

Education of caterers and consumers of chicken liver dishes to highlight the importance of thorough cooking to prevent ill health should continue [23]. In addition, in light of the background of ongoing education that has already occurred, caterers who fail to protect the health of their customers through inadequate cooking of chicken livers should face prosecution by the local authority for poor food hygiene. This is dependent on effective and rapid collection of environmental, microbiological and epidemiological information as evidence in support of prosecution. The outbreak investigation described in this paper collected exposure data as signed witness statements providing evidence that was used to successfully prosecute the caterer responsible for poor food hygiene practice.

## CONCLUSION

The result of the statistical analysis demonstrated that chicken liver pâté was the most likely cause of the foodborne outbreak of campylobacteriosis with a clear dose–response relationship having been established. The continuing need to raise awareness in caterers and the public of the risk to human health posed by undercooked chicken liver is evident. This requires the ongoing support of enforcement of food safety legislation by local authorities.
